# Key barriers and decision supports in revitalizing heritage buildings from investors’ perspective in China: A case study in Guangzhou, China

**DOI:** 10.1371/journal.pone.0311757

**Published:** 2025-01-24

**Authors:** Kailun Fang, Yifei Wu

**Affiliations:** 1 Guangzhou Urban Planning and Design Co., Ltd., Guangzhou, China; 2 School of Architectural Engineering, Shenzhen Polytechnic University, Shenzhen, China; Xiamen University Malaysia, MALAYSIA

## Abstract

In the decision-making process for investing in heritage buildings (HBs), various factors such as costs, interests, and tenancy terms influence investors decisions. Understanding the motivations of these investors can facilitate the involvement of social forces with diverse interests in adaptive reuse projects. This paper examines the primary barriers to revitalizing heritage buildings through adaptive reuse decision-making. The specific objectives of this study are as follows: i) To explore the barriers faced by investors in the adaptive reuse of heritage buildings. ii) To provide a comprehensive review of the factors influencing the adaptive reuse of heritage buildings, with a particular focus on developing countries. Through structured interviews with 19 investors and field research, three typical categories in the adaptive reuse decision-making process were identified: i) tenancy terms, ii) function, and iii) fire control problems. These findings indicate that vacancy is detrimental to the protection of heritage buildings, while rent plays a significant role in safeguarding them, as revealed by a correlation analysis using quantitative methods. Policymakers should better understand the expectations and needs of the public investors better to enhance support for optimal adaptive reuse decisions.

## 1. Introduction

An increasing number of cities take innovative approaches innovative approaches to address the issue of vacant or underutilized heritage buildings. Heritage buildings are a type of cultural heritage with architectural, aesthetic, historic, documentary, archaeological, economic, social, and even political and spiritual or symbolic values; However, their first impact is always emotional, as they are symbols of our cultural identity and continuity. HBs evoke a sense of wonder and carry various academic and aesthetic values [1–3]. Cities are implementing comprehensive strategies and policies to preserve and revitalize these buildings. By attracting tourists and generating significant revenue through tourism, heritage buildings contribute to the local economy and support various segments of society [4].

The adaptive reuse of vacant heritage buildings is being incorporated into their urban development and affordable housing plans, aligning with the principles of ’smart growth.’ By reusing heritage buildings in urban areas, these cities promote sustainability and smart growth initiatives, that focus on redeveloping inner cities to reduce urban sprawl [5]. Adaptive reuse provides an alternative to our throwaway culture by utilizing existing infrastructure and materials, making it a more sustainable option for construction projects [6].

HBs contribute significantly to the identity and character of our communities, serving as tangible connections to the past [7]. Many historic districts across the country are currently experiencing remarkable revitalization, with cities using their cultural landmarks as focal points for redevelopment efforts. However, the preservation and revitalization of HBs can be hindered by financial constraints, restrictive zoning and codes, contamination, and structural issues that pose challenges to their reuse.

While previous studies have examined barriers, general measurement methods like life cycle analysis, and technological proposals such as blockchain applications for closing material loops [8], there is limited research focusing on policies and actions at the micro level within the planning tools specifically tailored for developing countries. In order to facilitate monitoring activities and keep track of the conservation status of the HBs, adaptive use factors in the decision process are crucial to support decision-making processes for revitalization purposes. The new opportunities brought by digital tools can be of great help in this regard [9]. Although an increasing number of countries are supporting open HBs data and promoting its reuse, systematic digital systems including main decision factors for the revitalization of HBs are still lacking. Moreover, digital decision support systems focus on developed countries like Italy, France, and so on, but fail to focus on developing countries [10, 11]. The main problem in adaptive reuse projects is the random decision of the new function for heritage buildings without in-depth analysis which results in the waste of money for investors. Unfortunately, there is a lack of clear methodology for adaptive reuse decision-making of heritage buildings. It is mostly focused on environmental, physical, and functional aspects of heritage buildings, and there is less support for investment in heritage buildings. In sum, the research questions are as follows: 1) What are the main barriers of the adaptive reuse of heritage buildings for investors? 2) What are the main factors for investors in revitalizing heritage buildings in developing countries?

Accordingly, this research focuses on three objectives: 1) To explore the barriers of the adaptive reuse of HBs for investors; and 2) To provide a comprehensive review of the factors influencing the adaptive reuse of HBs and identify factors in decision-making for developing countries. These objectives aim to create a holistic understanding of adaptive reuse by offering both a detailed analysis of investor challenges and a broader perspective on how these challenges interact with other influencing factors in diverse contexts.

The structure of this research is as follows: Section 2 reviews the relevant literature. Section 3 describes the methodology used to integrate diverse fields of research. Section 4 presents the result of this research. Section 5 discusses the policy implication to solve main barriers. Section 6 summarizes the main conclusion, contributions, and limitations.

## 2. Literature review and theoretical framework

### 2.1 Adaptive reuse of HBs

Adaptive reuse of HBs is increasingly recognized as an important strategy for protecting many cultural heritages while promoting sustainable urban development. By repurposing historic structures for contemporary uses, adaptive reuse can also ensure the service life period of these buildings, integrating them into the modern function. However, this practice is not easy and has many challenges that require thoughtful consideration and other innovative solutions.

The revitalization of HBs plays a priority role in preserving HBs. Adaptive reuse not only ensures architectural integrity but also revitalizes urban environments by incorporating historical structures into the landscape. This integration is essential for maintaining the cultural identity of communities while simultaneously enhancing their economic and social vitality [12]. Effective revitalization, however, necessitates advanced restoration techniques. For instance, the application of alkoxysilanes in the restoration of decayed wood in HBs demonstrates how chemical treatments can stabilize and restore aged materials, ensuring that historic structures retain their authenticity while being adapted for new purposes [13]. Additionally, modern approaches such as scan-to-BIM facilitate the documentation and analysis of heritage buildings, enabling precise digital modeling and informed decision-making during the adaptive reuse process. The integration of technology in conservation underscores the potential for innovation in preserving heritage buildings [14]. Innovative partnership schemes have also emerged as essential mechanisms to address funding challenges, ensuring that HBs are repurposed in ways that benefit both communities and investors. These works highlight the intersection of preservation, sustainable development, and environmental impact, as evidenced by case studies in Hong Kong and Portugal [15–18]. However, existing research often falls short in addressing the potential tensions between preservation goals and the demands of modern urban development, leaving a gap in understanding how these conflicts might be managed.

Secondly, successful adaptive reuse projects can enhance visitor engagement by preserving the authentic character of historic buildings while adapting them to contemporary uses. This approach not only conserves cultural heritage but also fosters a sense of continuity and identity among visitors [19]. Communities can maintain a tangible connection to their past while adapting to contemporary needs [20]. While adaptive reuse offers numerous benefits, it also presents significant challenges. Organ (2020) identifies key obstacles such as funding constraints and the need for specialized conservation expertise, yet he also highlights the opportunities for adaptive reuse to enhance the sustainability and economic viability of historic sites. This duality reflects the broader tension within the field between the ideal of preserving heritage and the practicalities of modern development [21]. Adaptive reuse projects should focus on the stories and meanings associated with heritage structures. This approach emphasizes the importance of cultural significance in decision-making, ensuring that adaptive reuse aligns with the values of the communities it serves [22–26]. However, the challenge lies in balancing these narratives with the functional requirements of contemporary use, a balance that is often difficult to achieve in practice.

In short, the adaptive reuse of HBs is a complex and multifaceted approach that requires careful consideration of both preservation goals and contemporary needs. While it offers significant benefits in terms of cultural preservation, sustainable development, and social revitalization, it also poses challenges that must be critically addressed to ensure that these projects contribute positively to the communities and environments they aim to serve.

### 2.2 Barriers in revitalizing the HBs

Revitalizing HBs presents a challenge that involves a variety of technical and conceptual barriers. These barriers can significantly hinder successful conservation and adaptive reuse, strongly needing a thoughtful approach to ensure that historic structures are both preserved and adapted for modern use.

One of the primary challenges in this process is the tension between preserving the historical reliability of HBs and meeting modern functional requirements. Traditional conservation practices often emphasize the physical preservation of a building’s architectural integrity. However, this focus can sometimes come at the expense of the cultural narratives and values that are deeply embedded within these structures. When conservation efforts prioritize the physical aspects alone, there is a risk of creating a disconnect between the building’s historical significance and its modern function, making it difficult to negotiate both preservation efforts with the cultural and social needs of the community [27].

Another significant barrier is the challenge of ensuring fire safety in HBs. Implementing modern fire safety standards in buildings not originally designed for such requirements presents considerable difficulties. The need to preserve historic materials and architectural features often conflicts with the necessity of incorporating fire-resistant elements, creating a dilemma for conservationists. Torero’s methodological approach highlights the importance of developing fire safety strategies that are sensitive to the historical context of the building while providing adequate protection against fire accidents. However, finding a balance between these needs is complex and often results in compromises that can either undermine the building’s authenticity or leave it vulnerable to fire-related risks [28].

The structural integrity of HBs, particularly those constructed from unreinforced masonry, poses yet another significant barrier. The inherent limitations of these structures complicate efforts to retrofit them for seismic resilience. Conservationists must balance the need for structural reinforcement with the desire to preserve original materials and construction techniques, a task that is fraught with technical and ethical challenges [29].

In addition to these technical challenges, the decision-making process in the adaptive reuse of HBs has difficulties, especially when it comes to selecting appropriate utility functions. Fedorczak-Cisak et al. (2019) discuss the challenges of applying multi-criteria decision-making approaches to HBs, noting that the need to respect historical significance often clashes with the demands of modern use. This tension is particularly pronounced in cases involving multiple stakeholders with differing priorities, making consensus difficult to achieve [30].

Fire prevention presents additional challenges, particularly in HBs where there is a need to make safe practices without compromising the building’s historical integrity. Kincaid (2022) discusses various approaches to fire prevention, emphasizing the importance of strategies that are both effective and sensitive to the historical context. However, the requirement for sensitivity can limit the effectiveness of fire prevention measures, creating a barrier to the safe and sustainable use of buildings [31–34].

Despite the extensive examination of specific barriers in the revitalization of HBs, such as the tension between preserving historical authenticity and meeting contemporary functional requirements, fire safety, and structural integrity, there is a noticeable lack of comprehensive studies that integrate these challenges into a unified approach. Existing research often treats technical and conceptual barriers as separate entities, overlooking the interdependent nature of these challenges and their collective impact on the successful adaptive reuse of HBs. Moreover, while the decision-making process in adaptive reuse has been addressed, particularly in selecting appropriate modern functions, there is limited research on effectively balancing the competing priorities of different stakeholders within a single framework. Although the need for a approach that respects both historical significance and modern use is recognized, the practical application of such an approach still needs to make better.

To address these gaps, future research should focus on developing a holistic framework that synthesizes these various challenges, offering practical solutions that account for the relevance of technical and conceptual barriers. Such a framework will provide valuable guidance for practitioners and policymakers, ensuring that revitalization efforts are both sustainable and sensitive to the cultural heritage of historic buildings.

### 2.3 Conceptual framework

Investors in HBs, as identified in the literature, encompass a diverse group of stakeholders, including government agencies, private owners, financial institutions, and real estate developers. Their involvement is not merely transactional but is deeply intertwined with the broader objectives of cultural preservation and economic sustainability [35–38]. Each of these entities approaches investment with distinct motivations and responsibilities, which can significantly influence the outcomes of restoration and adaptive reuse projects.

To be more precise, in cases where the government is the building owner, it often directly invests in both the renovation and operation of the historic structure. The government acts as both the owner and the investor, aligning public preservation goals with the broader socio-economic benefits of protecting HBs. This direct involvement ensures that the cultural significance of the building is preserved while also supporting community development.

In contrast, when the building is privately owned, the owner may choose to transfer the right to use the property to another one, who then assumes the role of the investor responsible for restoration and operation. This introduces a separation between ownership and investment, often leading to different priorities between the two. The investor, driven by the need to achieve a return on investment, may focus on maximizing the building’s economic potential, sometimes at the expense of its history.

Further complicating this landscape is the scenario where the investor, after renovating the building, transfers its operation to a third party, typically a tenant. The tenant is the investors. In short, the spectrum of investors in historic buildings includes government bodies, private heritage building owners, and financial organizations, with each playing a distinct role depending on the ownership and operational structures in place. The critical challenge lies in balancing the cultural, social, and economic objectives across these varied stakeholder groups to ensure that historic buildings are preserved in a way that respects their heritage while also adapting them for modern use. This complexity necessitates a nuanced understanding of the motivations and limitations of each type of investor to guide successful revitalization efforts [35–38]. The standpoint of these "investors" regarding adaptive reuse is typically pragmatic and business-oriented. The primary motivation for participating in an adaptive reuse project is often financial. These investors are usually more concerned about the return on investment from implementing the adaptive reuse project, as well as the well-being of the users and the community. As risk bearers, they critically assess the potential profitability (or at least breaking even) of reusing an existing building before venturing into such projects. Prior to investing in an adaptive reuse project, investors typically explore the available financial incentives and carefully consider the associated regulations [39].

**Fig 1:**
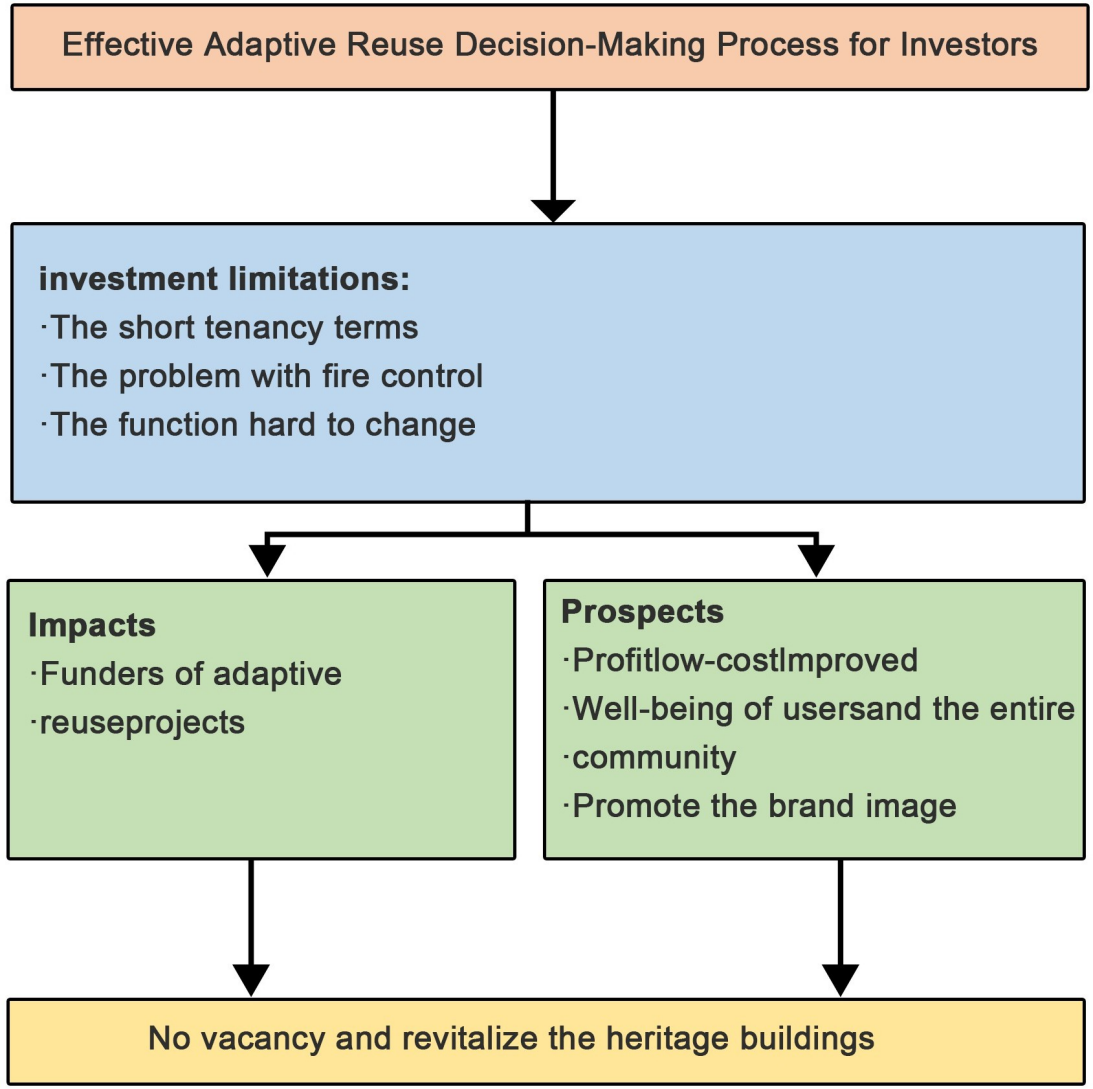
Conceptual framework for an effective adaptive reuse decision-making process for investors.

To ensure effective investor participation in an adaptive decision-making process, this study enabled the development of a theoretical framework for the integration of collaboration into the adaptive reuse decision-making process including the key essentials of collaboration: (i) investment limitations; (ii) prospect; and (iii) impacts. Investment limitations in the adaptive use of old buildings refer to the constraints and challenges that arise when repurposing existing structures for new functions. While adaptive reuse offers numerous benefits, stakeholders must consider financial aspects and potential obstacles that could affect the project’s feasibility. On the other hand, the prospect of adaptive use in old buildings involves identifying potential opportunities and advantages associated with transforming existing structures for new purposes. Stakeholders evaluate various benefits and positive outcomes that can be achieved through this transformation. Additionally, the impact of adaptive use in old buildings considers the effects and consequences of converting existing structures for new functions on the community and environment. Understanding these potential impacts is essential for making informed decisions and ensuring that the adaptive reuse project aligns with sustainable and community-oriented goals (See Fig 1).

## 3. Methodology

### 3.1 Study context

#### 3.1.1 Legal protection of cultural heritage in China

In China, heritage building legal is integrated into the broader framework of cultural heritage legal protection laws. The enactment of the national HB protection law in 1982 marked a significant milestone in establishing a modern legal regime for heritage buildings. Since then, the law has been amended four times: in 1991, 2002, 2007 and 2013, respectively [40]. According to the policy, alterations to the interior structure of a HB are generally prohibited. Additionally, some HBs’ designated for residential use may not be suitable for other functions.

#### 3.1.2 Case selection

Guangzhou has been actively engaged in top-level design related to cultural heritage protection legislation, leveraging legal construction to ensure and promote the protection and inheritance of cultural heritage. Evidenced through archaeological artifacts and historical landmarks, archaeological artifacts and historical landmarks reflect the core elements of Cantonese culture, including humanism, pragmatism, industriousness, and originality. These fundamental cultural aspects are evident in various domains such as architectural styles, traditional practices, landscape design, commercial endeavors, spiritual beliefs, and artistic forms. These fundamental cultural aspects frequently manifest themselves across domains such as architectural styles, traditional practices, landscape design, commercial endeavors, spiritual beliefs, and various artistic forms. The profound implications, multifaceted nature, and unique characteristics of Cantonese culture are expressed through linguistic traditions, musical compositions, theatrical performances, written arts, visual arts, poetry, architectural designs, miniature horticulture, handcrafted creations, customary practices, and culinary traditions [41].

Guangzhou has implemented a wide range of policies that focus on cultural heritage. The HB protection policy in Guangzhou was enforced in 2013, followed by the implementation of the HB revitalization and utilization policy in Guangzhou in 2019. Despite being a leading city in the country, Guangzhou encounters difficulties when it comes to revitalizing heritage buildings. Additionally, being a prominent first-tier city in China, Guangzhou attracts numerous investors interested in heritage buildings [42]. Therefore, the selection of Guangzhou as a case study for this research was based on these factors.

Three activation and utilization modes had been formed in Guangzhou: (1) Government trusteeship and enterprise capital. Guangzhou adopted policy guidance, financial assistance, simplified procedures, rent reduction, and other methods and implemented the socialized trustee of government supervision and enterprise operation. For example, Wanmu Grass Hall is entrusted to the Yuexiu District Wende Culture Chamber of Commerce to manage and be used as a platform for culture research and display. (2) Government coordination and thematic museum. Guangzhou promotes the construction of special museums and exhibition halls for cultural relics with clear property rights. (3) Government support and social forces model. Guangzhou has introduced social forces to actively explore effective and feasible rational utilization models of cultural relics buildings such as exhibitions, cultural and creative industries, tourist attractions, and education bases. This research focused on all modes of revitalization cases.

### 3.2 Data collect

#### 3.2.1 Structured interview

In this research, qualitative data was collected through face-to-face, structured interviews with 30 investors in Guangzhou. The time period of interviews is from 1^st^ January 2023 to 23^rd^ May 2023. The owners of the HBs included private, government, and state-owned enterprises in Guangzhou. These investors who operated heritage buildings had converted their historical buildings into various establishments, such as boutique lodgings, company offices, cafes, hotels, and canteens. A total of 30 operators were included in the study population, and we reached out to all of them through invitation messages, followed by telephone calls. Out of the operators contacted, only 19 agreed to participate in the interviews (as shown in Table 1), while the others declined due to personal reasons. The interviews took place at the respondents’ offices, at a time convenient for them, and lasted between 60 and 90 minutes. The interviews were conducted in Chinese and later transcribed verbatim. The transcripts were then translated into English by the researchers. Two researchers conducted the interviews, and the sessions were recorded using interview forms. To ensure the validity of the outcomes, we deliberately employed a purposive sampling method, which was essential given the specificity and complexity of the research subject—investment decision-making in the adaptive reuse of HBs. The participants were carefully selected based on their direct involvement in HB investments, ensuring that they possess the requisite expertise to provide meaningful insights. This targeted approach, though involving a smaller sample size, allows for the collection of highly relevant data, which is often more insightful and contextually rich than larger samples that may lack the same level of domain-specific knowledge. While the number of participants (19) may seem limited, this smaller group represents a highly specialized subset of stakeholders whose opinions and experiences are crucial to understanding the nuanced challenges of HB revitalization. The qualitative nature of this research further supports the sample size, as the objective was not to generalize findings across a broad population, but to deeply explore the intricate decision-making processes of expert investors in this field. In such cases, smaller but more focused samples often yield higher validity and reliability, as they avoid the dilution of specialized knowledge that can occur with broader, more general sampling.

**Table 1:** Interviewees’ information.

Number	Heritage buildings code	Ownership	Level	Investors	Past Functions	Existing functions
**1**	HB-1	Non-state-owned	A Major Historical and Cultural Site Protected at the National Level	Company	Residence	Retail, office
**2**	HB-2	Non-state-owned	A Major Historical and Cultural Site Protected at the National Level	Company	Commerce	Retail, office, light catering
**3**	HB-3	Non-state-owned	A Major Historical and Cultural Site Protected at the National Level	Company	Dwell	Retail, display, light catering
**4**	HB-4	Non-state-owned	Historical and Cultural Sites Protected at the City Level	Private	Temple of family	Stone museum
**5**	HB-5	State-owned	Historical and Cultural Sites Protected at the Provincial Level	Company	Residence, office	Museum
**6**	HB-6	Non-state-owned	Historical and Cultural Sites Protected at the County Level	Company	Residence	Exhibition
**7**	HB-7	State-owned	Historical and Cultural Sites Protected at the City Level	Company	Education	Museum
**8**	HB-8	State-owned	Historical and Cultural Sites Protected at the City Level	Company	Residence	Office
**9**	HB-9	Non-state-owned	District registered cultural sites	Company	Temple of family, residence	Homestay
**10**	HB-10	Non-state-owned	Historical and Cultural Sites Protected at the City Level	Company	Residence	Office, business, canteen
**11**	HB-11	State-owned	A Major Historical and Cultural Site Protected at the National Level	Company	Ancestral temple	Exhibition
**12**	HB-12	State-owned	Historical and Cultural Sites Protected at the City Level	Company	Academy of classical learning	Cultural activity
**13**	HB-13	State-owned	District registered cultural sites	Research Association	Family school	Office
**14**	HB-14	State-owned	Historical and Cultural Sites Protected at the Provincial Level	Company	The Silk Trade Hall	Exhibition
**15**	HB-15	Non-state-owned	Historical and Cultural Sites Protected at the City Level	Primary school	Academy of classical learning	Primary school
**16**	HB-16	State-owned	District registered cultural sites	Company	Staff quarter	Office tea room and other business
**17**	HB-17	Non-state-owned	Historical and Cultural Sites Protected at the City Level	Company	Ancestral temple	Intangible cultural heritage activities
**18**	HB-18	State-owned	Historical and Cultural Sites Protected at the Provincial Level	Museum	Temple of family	Exhibition
**19**	HB-19	State-owned	District registered cultural sites	Company	The Pawn	Restaurant

Additionally, the structured interview process contributed significantly to the consistency and comparability of the data. By employing a standardized set of questions, we minimized interviewer bias and ensured that key themes were systematically explored across all interviews. This structured approach not only enhanced the depth of the qualitative data but also allowed for the identification of patterns and commonalities among participants, thereby strengthening the overall robustness and credibility of the findings.

To guide the interviews, we developed an interview guideline based on suggestions from existing literature that aligned with our conceptual framework, research questions, and objectives. The guideline comprised of ten questions focusing on factors that define boutique hotels, the aims and motivations for adaptive reuse, the functions and number of spaces previously and currently used for adaptive reuse, as well as the risks and limitations associated with adaptive reuse.

Interviewees were asked the same basic questions in the same order, thereby increasing the comparability of responses. Interview questions were derived from several sources and considered indicators such as HBs value, how consultation works, maintenance difficulties and compensation, ownership, environmental benefits, and contribution to the local economy, cost of renovations, and limitations of HBs were also considered.

The questions had two parts. The first one is to classify the basic information of the building. The second parts were designed to reach the research objective. The interview questions are as follows:

1. What is the status of your revitalized heritage property rights? What is the current utilization of the HB? Who is responsible for the protection and management of HB, and who is responsible for the safety of the HB?
2. What was the initial investment before the HB was officially activated? What were the sources of upfront investment funds?
3. What is the status of the daily operation of the HB and the capital flow?
4. What do you think are the difficulties in the revitalization of the HB? (including fire protection, lease terms, repair requirements, etc.)
5. In what way was the subject of the HB recruited and activated? What are the pros and cons of this approach (entrustment, open competition, etc.)? What is the specific process?
6. To further enhance the sharing of HB information, we plan to use the existing government platform to provide relevant information about idle HB in the near future. In your opinion, what necessary and important HB information needs to be displayed and explained to the public?
7. What other functions do you hope the cultural relics information platform can have besides providing information on idle HB?
8. In what ways does the heritage information platform provide guidelines for public use? (policies and regulations, excellent cases, operational guidelines, etc.)

Using multiple case studies can enhance external validity and reduce the risk of observer bias, leading to the development of more robust and testable theories compared to relying on a single case study [43]. Criteria were established to ensure that the selected cases were relevant to the research objectives. First, to align with the definition of investors, the cases had to involve individuals or entities that have invested in and revitalized HBs. Second, the investors chosen for the study should have demonstrated success in revitalizing HBs. The following criteria were used to select interviewees: they must have a minimum of five years of experience in HB investment, and they should hold a key leadership position, which would give them a comprehensive understanding of the investment and its processes. This approach is consistent with common selection criteria in studies aimed at assessing entire organizations based on empirical evidence from a limited number of respondents [44, 45].

The research employed a systematic methodology to investigate the adaptive reuse of HBs. Initially, a comprehensive database was established through an extensive literature review and data collection, forming the foundation for identifying key factors influencing HB reuse. Following this, a field investigation was conducted, including site visits to assess the physical state and outcomes of HB revitalization projects, focusing on structural integrity, design changes, and current usage. Typical samples for interviews were selected through government connections and snowball sampling, ensuring a diverse and representative set of cases. An initial introduction meeting was held with stakeholders to explain the research purpose and interview process, ensuring informed participation. Structured interviews were then conducted, with consent, allowing interviewees to provide detailed insights while maintaining focus on the research objectives. The data collected from site visits and interviews were meticulously documented and analyzed through transcription, coding, and comparative analysis, culminating in a synthesis that provided a comprehensive understanding of the challenges and factors influencing HB adaptive reuse, particularly from the perspective of investors and decision-making processes. Initially, 30 potential participants were contacted for structured interviews. Out of these, 19 participants agreed to be interviewed. The research needed to revise the manuscript to accurately reflect this distinction, ensuring clarity in the description of the data collection process.

#### 3.2.2 Document analysis

According to the official report of *The Assessment of Heritage Conservation Status* conducted by the third institution, the HBs could be evaluated for their interior structure, interior condition, façade status, and so on. The assessments gave marks (1–5) for protection status, in which 5 means perfect and 1 means poor.

### 3.3 Data analysis

This research proposes an innovative approach using curve-fitting method to examine the relationship between vacancy and rent.

Deductive content analysis was performed, with similar ideas grouped together to form code, basic theme, organizing theme, and global theme for making valid inferences from data to their context, with the goal of providing knowledge, new insights, a representation of facts, and a practical guide to action to achieve a condensed and broad description of the phenomenon. Each category is named with content-specific words. Subcategories with similar events and incidents are grouped together to form categories, and categories are grouped together to form main categories [46].

## 4. Results

### 4.1 The overall attitude toward investors towards HB revitalization

The existing functions were non-profit functions such as residence and museum, and for-profit functions such as office and business are very important for activation. Most of the function of HBs was for non-profit use and little was for profit use.

**Fig 2:**
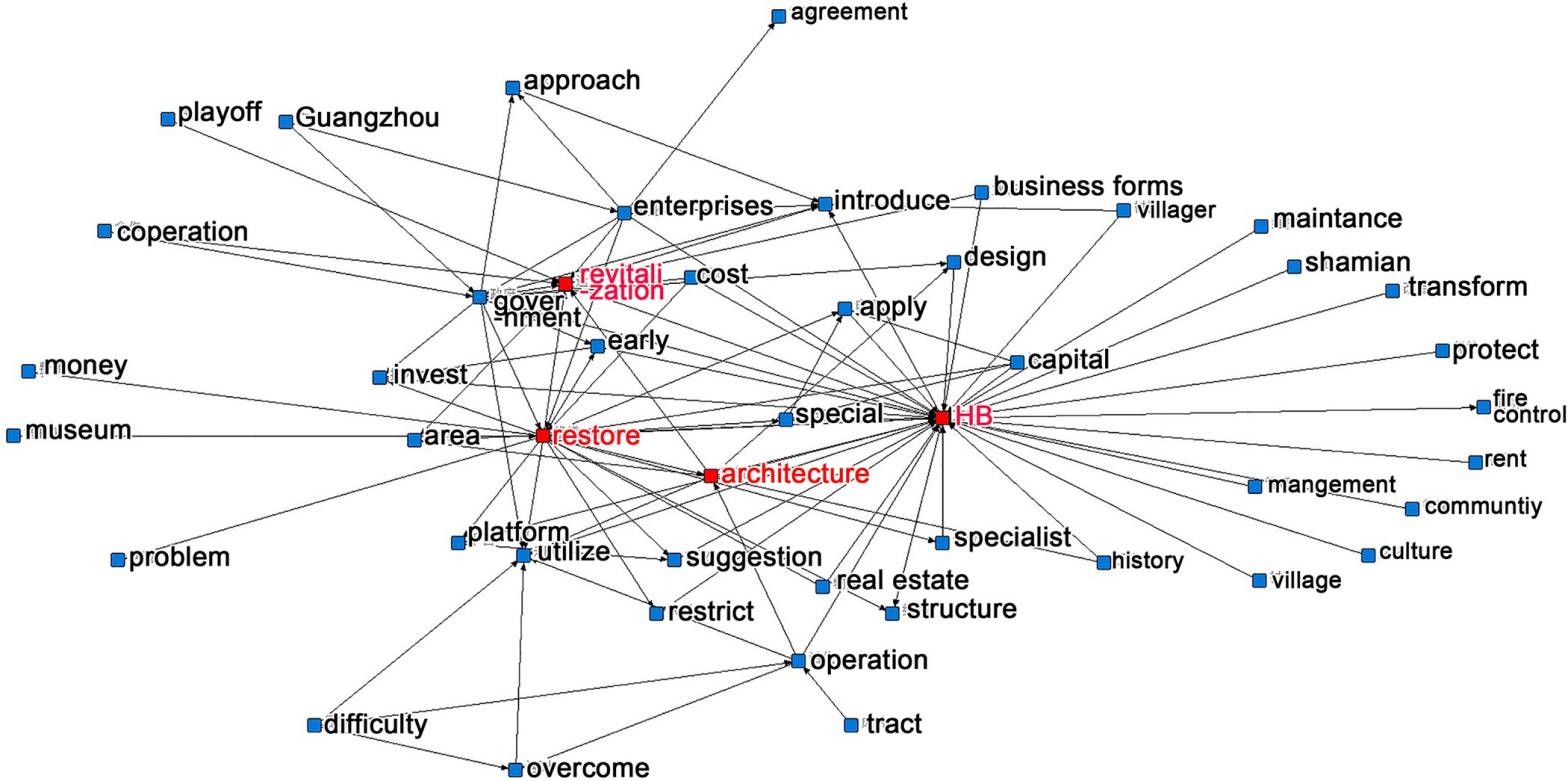
Semantic analysis graph.

With the help of NetDraw, a semantic analysis tool in ROST-CM6, the intensity of semantic associations in HB revitalization was identified. The co-occurring terms were automatically classified into four clusters through a correlation operation, generating a semantic network map for HB revitalization (Fig 2). Building operations and building repairs were two major concerns. The function directly correlated to HB revitalization but people responsible for HB revitalization did not know how to consult before revitalization, resulting in the misleading of function chosen.

Furthermore, the implementation of a narrative approach was equally crucial in enhancing the function of HBs. Adopting a narrative approach had the potential to revolutionize conservation theory and empower dedicated volunteers who are passionate about preserving our HBs. Narrative transcended from being perceived as a burdensome task and instead becomes an exciting journey of exploration. This transformation has the capacity to profoundly impact our understanding of ourselves, both within the professional and non-professional realms.

Only a few agreed on the environmental benefits and the contribution of listing to the local economy, suggesting that most interviewees were unfamiliar with the sustainable development concept. Although many individuals responsible for HB revitalization currently reside in or work on their listed properties, only a few indicated that they actually renovated their HBs. The high cost of labor and materials required for renovations is a significant barrier (Table 2).

**Table 2:** The overall attitude for revitalization related to interview questions and results.

Indicators	Measures	Evidence derived from interviewees’ comments
Planning practice	Consultation	Did not know how to consult before revitalization, resulting in the misleading of function chosen
	A willingness to repair the building	Reside or work on their listed properties
		Currently reside or work on their listed properties
		The cost of labor and materials needed for renovations are expensive
Implementation	Function selection	They were a good investment if the HBs were used as retail, and catering function can attract more people to visit and spend money than the museum or facility function, especially for the HBs located in the central district
	Method	The implementation of a narrative approach is equally crucial in enhancing the function of HBs
		Narrative transcended from being perceived as a burdensome task and instead becomes an exciting journey of exploration
	Benefit share	The environmental benefits and the contribution of listing to the local economy

### 4.2 The vacancy was not good for protection, whereas rent played a crucial role in safeguarding heritage buildings

**Fig 3:**
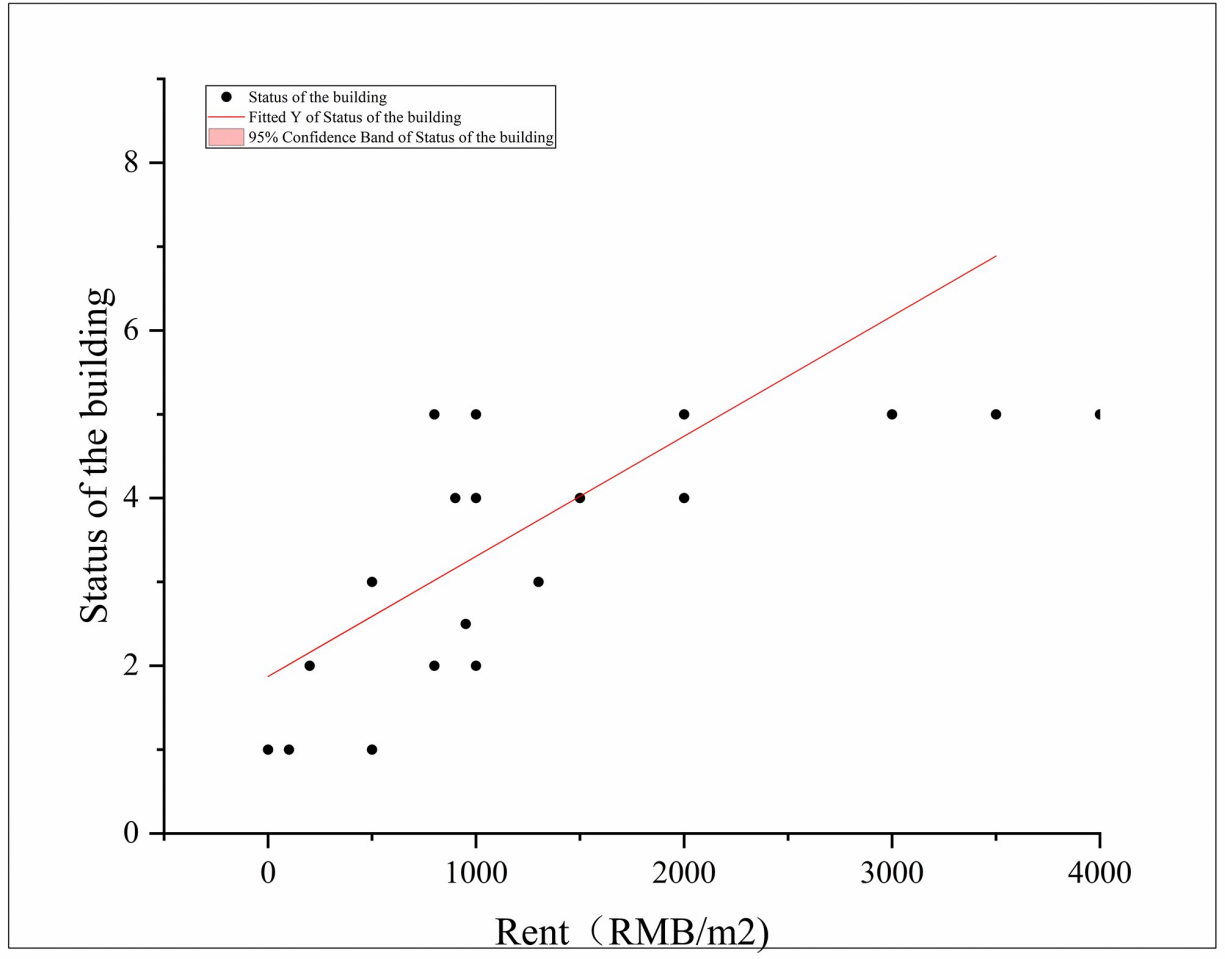
The correlation analysis between rent and status of HBs.

The preservation of a HB was crucial to maintaining its integrity and preventing it from falling into disrepair. When HBs were left vacant, they were at a significantly increased risk of damage [47, 48]. Small damages might have gone unnoticed without people living or working inside, and over time, these damages could accumulate and lead to significant deterioration. To prevent the potential destruction of vacant HBs, stabilization and mothballing techniques were employed as temporary measures to protect it from demolition. However, the most effective way to breathe new life into HBs was by attracting people to utilize them. Adaptive reuse, which involved repurposing buildings that had outlived their original purpose, was a key strategy in achieving this goal. By repurposing HBs, they could be preserved, while simultaneously addressing issues such as urban blight and promoting social change. Generally speaking, HBs without collapsed repairs or water leaks that threaten the indoor environment were considered to be better preserved. It was worth noting that the protection status of HBs often correlates with their rental value. When the rent for a HB was high, it indicated that it was better protected and valued for its historical significance. This recognition of their worth led to increased efforts in preserving and maintaining these buildings. In summary, preventing HBs from remaining vacant was essential to their preservation. By attracting people to use and occupy these buildings, the risks of damage and deterioration can be minimized. Adaptive reuse serves as a means to repurpose HBs, preserving their heritage while also contributing to urban revitalization and social transformation. The higher rental value of HBs was indicative of their better protection status, emphasizing the importance of valuing and safeguarding these invaluable architectural treasures (See Fig 3).

### 4.3 Main barriers to revitalize HBs

**Fig 4:**
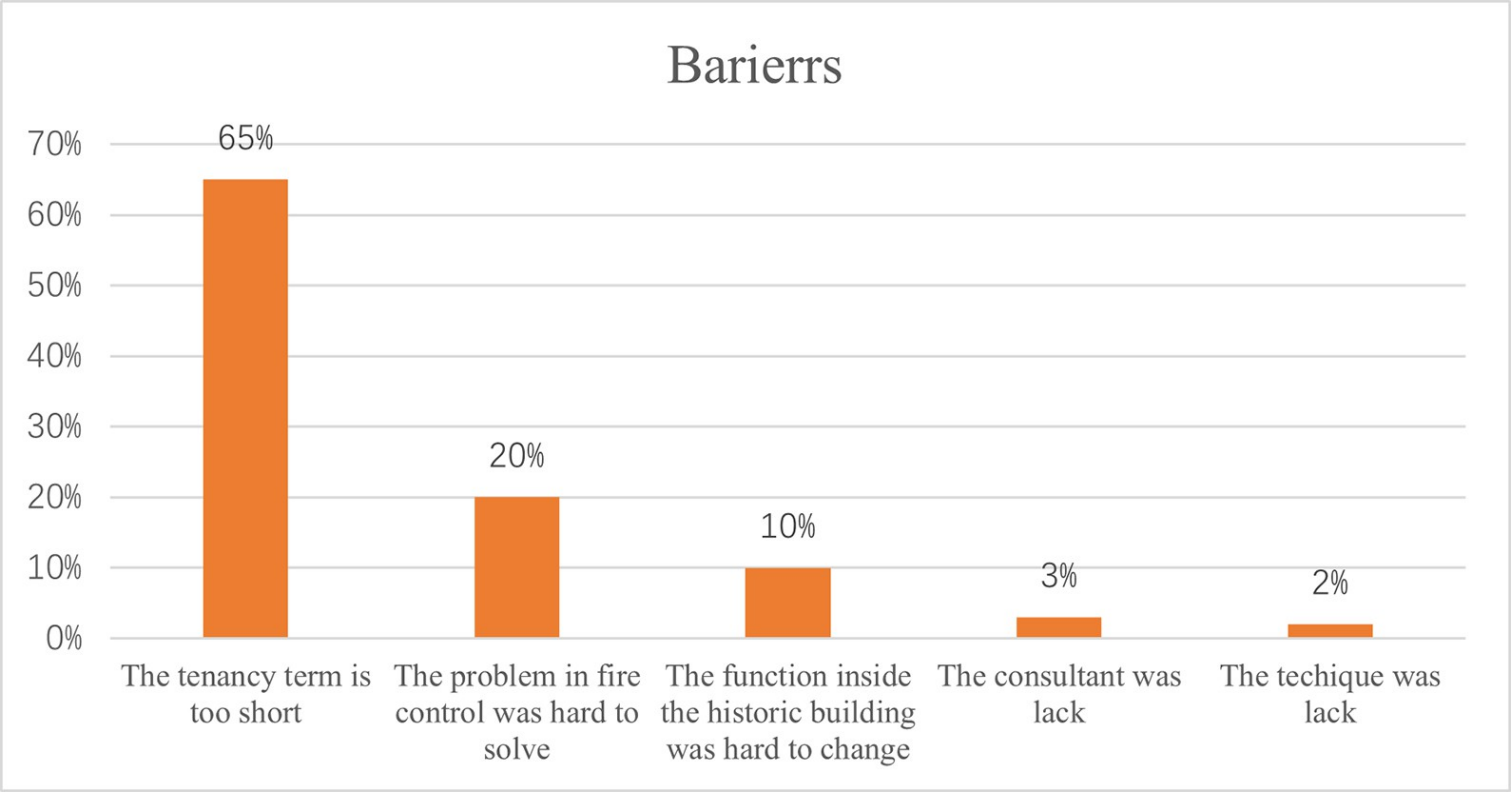
The limitation percentages.

The barriers included five perspectives: (1) The tenancy term was too short; (2) Fire control was hard to solve; (3) Changing the function inside the HB was challenging; (4) There was a lack of consultancy; (5) There was a lack of technical expertise. Since the top three barriers exceeded a 10% threshold in terms of percentages, this section primarily focused on elaborating upon these three significant obstacles (See Fig 4).

#### 4.3.1 The tenancy term was short

The ownership of HBs in Guangzhou could be divided into two categories: privately-owned and government-owned. Privately-owned HBs included private property (person-ownership, private sector ownership, and collective ownership). Due to the HB’s inherent values and its status as an important urban landmark, it was considered a cultural resource to be enjoyed by the public at large and protected for future generations. Therefore, the government and the public are also their stakeholders. The government had the responsibility of conserving these HBs in a way that did not contravene their private ownership, while ensuring their safeguarding and contribution to the overall quality of the community. Government-owned HBs involved local and central government, local government-owned sectors, and central government-owned sectors. For the government-owned properties, the policy in Guangzhou stated that they could be rented for five years.

Around 65% of interviewees were complaining about the short tenancy terms (Fig 4). In this context, the government often owned many HBs and leases them to investors for a relatively short duration of five years. Typically, investors dedicate approximately three years to restoring the historical structure, leaving them with just two years to establish and operated a new enterprise within the building. This abbreviated timeline could present difficulties for investors, as it limited the amount of time, and they must recoup their investment and generate profits. Moreover, considering the significant effort and resources required for restoring a HB, the remaining two years might not be sufficient for investors to fully capitalize on their investment and established a sustainable business. It was important to note that these leases’ specific terms and conditions might vary on a case-by-case basis and could be subjected to negotiation between the government and the investors. Nonetheless, the relatively short tenancy term in China’s HB rental market presented unique challenges for investors seeking to utilize these HBs for commercial purposes.

Another premise was that the return had a reasonable cycle. It generally took 8 to 12 years to recover the capital after the restoration of a HB. The location of the HB, the average rent level, the length of the lease, and how to recover the cost were important factors to consider. One HB in was requisitioned as a resettlement house and kindergarten and remained idle for decades before being renovated. After 3.5 years of renovation, the building was successfully leased in 2020. Later, the investors recovered the house and began operating it independently. The initial process was lengthy and costly, and it took 8 years for investors to see returns from the revitalization of cultural relics buildings, making it difficult to achieve immediate profits. The rent period played a crucial role in the HB revitalization for investors.

*The government has invested 20 million yuan in the renovation of HB-17, but it still needs to purchase more than 5 million yuan for operation and maintenance services every year (I7*).

*After the government completed the basic repair work of HB-4*, *the social forces still needed to invest 8 million yuan for the repair*, *which greatly exceeded the budget*. *After the government invested 3 million yuan in the early stage of renovation*, *the HB-12 still has problems such as rainwater flooding and roof leakage and needs regular maintenance every year (I4*).

Despite the investors’ push for extending the tenancy term, the owners of the HBs were hesitant due to rental risks. Their main concern revolved around the potential future increase in the tenancy price. If they leased the property to the investors for an extended period, they would miss out on the opportunity to earn a higher rental income. Additionally, shorter tenancy terms could allow them to attract more favorable investors (See Table 3).

**Table 3:** Analysis of short tenancy terms.

Code	Basic theme	Organizing Theme	Global Theme
Upfront investment money	Input-output relationship	The tenancy term was short	Barriers to revitalizing HBs
The recovery period is long			
Low profit			
Lack of repair money			
Difficult to negotiate	Difficulty of renewal		
Afraid to be driven away			

#### 4.3.2 The function inside the HBs was hard to change

Since buildings’ function was crucial for successful revitalization, the investors like to operate in other functions like the hostel or restaurant. The interview results supported the facts that the function directly correlates to HB revitalization. Almost 45% found they were a good investment, if the HBs were used as retail, and catering function can attract more people to visit and spend money than the museum or facility function, especially for the HB located in the central district. An 80% agreed that retail and catering establishments offer tangible benefits to visitors, such as shopping opportunities and dining experiences, which often have a broader appeal and allure for the general public. One case was that HB-3 cannot use open flames, which had discouraged some catering practitioners. Obtaining a standard food license was difficult, so most investors only secure a business license without the ability to use open flames. The so-called business certificate referred to a temporary commercial certificate. When a building with a residential designation seek to engage in commercial activities, the first step was to obtain this business certificate. Only after receiving temporary commercial used approval from the local authorities can the business be registered with the Industrial and Commercial Bureau to obtain a full business license. These restrictions might also explain why HB-3 was still predominantly occupied by retail shops, coffee shops, and light catering outlets that do not require open flames. It is worth mentioning that the specific dynamics and preferences of each location can vary, and there may be cases where museums or facility functions in HBs enjoy significant popularity. Nevertheless, the general trend suggests that retail and catering functions tend to have a broader appeal and greater potential to attract visitors and generate revenue, especially for HBs situated in central districts.

In spite of the crucial role that function played in the process of revitalization, investors often encounter restrictions that prevented them from altering the interior structure of HBs, or they face considerable challenges when attempting to transition to a new function. A notable circumstance arose in cases where certain HBs were originally designed for residential purposes, rendering them unsuitable for accommodating alternative functions. As a result, the imperative for change within these HBs became increasingly apparent. The inherent dilemma of reconciling historical significance with contemporary utility compelled a shift in their usage (See Table 4).

*The investor hoped that the HB-8 would be transformed into an educational institution and needed to remove part of the pillars to obtain more space, but changing the internal space of HBs was not allowed, so the investor gave up and the HB-8 was left idle (I8*).

**Table 4:** Analysis of interior function of HBs.

Code	Basic theme	Organizing Theme	Global Theme
Structure could not be changed inside the HB	The compatibility of interior spaces and new functions	The function inside the HBs was hard to change	Barriers to revitalizing HBs
New function was not fitted into the old building			
Reversible repair was needed in the HB but not every designer knew this	Lack of new technology that could change the interior space without damaging the original structure of cultural relics		
The interior decoration should not harm the noumenon of buildings			

#### 4.3.3 The problem with fire control was hard to solve

HBs hold immense cultural significance; however, they posed a multitude of challenges when it came to fire safety. For example, in addition to the cultural display function of HB-3, it also hoped to configure as a coffee shop, but the current situation made it difficult to apply for a business license because the fire would be hard to control when this function comes to be, as the policy of coffee shop license belonged to the catering license which needs strict fire control standards. These buildings often struggled to meet the stringent fire control requirements mandated for new constructions, making it difficult for investors to determine the most suitable approach for renovating them. The architectural features, materials, and layout that contributed to their historical value may not align with modern fire safety standards. One case was that HB-7 faced challenges in meeting modern fire protection requirements, and the main relevant departments managed the situation through an approval and filing process. The government indicated that obtaining a fire safety certificate was not possible for this building. However, they requested that daily improvements be made to the fire facilities, such as installing a micro fire station and other necessary equipment. Additionally, they emphasized the importance of enhancing daily fire management through regular inspections, fire safety training, and similar measures.

*Coffee shop could be a great way to reach out to a larger audience and attract new catering clients. It would be clean with no fire without gas stove (I3*).

The national guidelines for HB revitalization and utilization stated suggestions on the use of HBs, including business services: small hotels, inns, homestays, shops, tea rooms, traditional craft workshops, and other business service places, to play service functions under the premise of ensuring safety. According to the national policy about encouraging the social capital to revitalize the HBs, during the period of management and use, HBs could be used to open public cultural places such as museums, exhibitions, art galleries, rural libraries, local culture centers and special cultural activity centers, and tourism and leisure service places, such as homestays, inns, tea houses, and so on. But in the implementation, there was a lack of supporting policies for functional business procedures.

Preserving the historical integrity of these buildings while ensuring adequate fire safety measures can be a complex task. Balancing the imperative to protect historical structures from the devastating impact of fires with the need to maintain their authenticity poses a delicate challenge. Investors are confronted with the dilemma of how to renovate HBs effectively, mitigating fire risks without compromising their cultural and architectural integrity. Successfully achieving this goal demands requires a comprehensive understanding of both fire safety regulations and historical preservation techniques [28].

## 5. Discussion and policy implication

**Fig 5:**
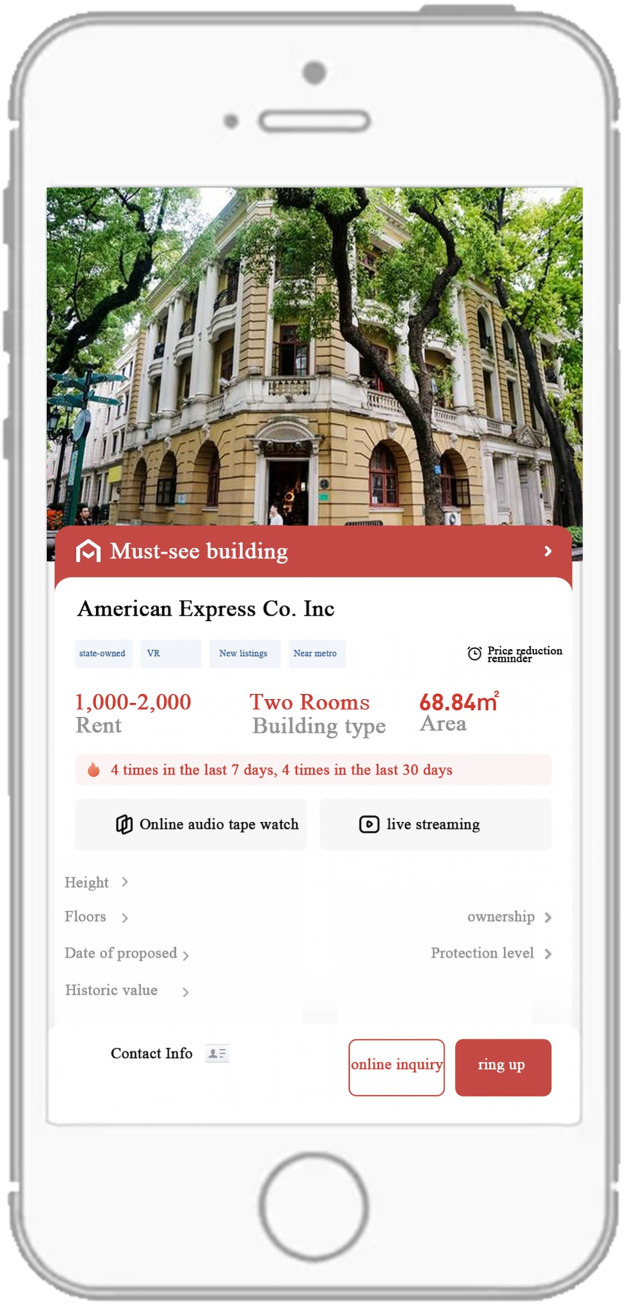
The example of the system.

Firstly, there were still few guidelines for content operation, and it was recommended to set up application procedures and management consultants. This platform should tell you how to find information when you have problems with repairs, and when you have problems with your business license. The platform should also provide the restoration cost of the cultural relic building, the restoration time, the functions suggested by experts, and the cultural stories the building carried. Moreover, it should encourage high-value HBs investors to make money to improve the surrounding public space if they have lower rent because of the policy discount (See Fig 5).

Hope information on the website had included: (1) Detailed information of the ownership; (2) Identify important buildings around to facilitate enterprises to estimate the flow of people; (3) Detailed introduction of stories behind HBs; (4) The rent should be marked as "reference rent", leaving some room for negotiation; (5) It was proposed to install a cost estimator to calculate the actual cost after the policy discount; (6) Corresponding preferential policies (I2).

Secondly, fire control should be case by case. Although the national guidelines for HB revitalization and utilization stated recommendations that include small hotels, inns, homestays, shops, teahouses, traditional craft workshops and other business service places to play service functions under the premise of ensuring safety, in fact, it is difficult to use for light catering such as homestays and teahouses. Due to the failure to pass the fire inspection and acceptance, HBs struggle to meet the standard of a homestay; there is only one homestay in Yuexiu District, and the social forces introduced by one village in Huadu District can only use the HBs as public facility at present. Because it is difficult to apply for a light catering business license, the activation force of cultural relics such as HB-3 can only be made of prefabricated products. The operation of social forces was subject to many restrictions, and it is difficult to legalize business forms such as light catering that can retain tourists and increase tour time and improve the actual benefits created by the activation and utilization of HBs. If the restrictions such as fire inspection and acceptance, and the application for business licenses for residential renovation companies, homestays, and light catering can be opened up, it will play a positive incentive role for social forces to participate in the activation and utilization of HBs.

Thirdly, it was essential to approach each project involving fire control with a single, dedicated discussion among the expertise of consultants and professionals specializing in heritage conservation and fire safety. Engaging expert consultants and professionals who specialized in heritage conservation and fire safety can prove invaluable in navigating this complex task. In recent years, there had been advancements in fire safety technologies and practices that cater specifically to HBs. These advancements encompass specialized fire suppression systems, improved evacuation plans, and fire-resistant treatments for historical materials. By remaining up-to-date with these innovations and collaborating with experts, investors can explore viable options for renovating HBs while addressing fire safety concerns. Ultimately, striking the right balance between preserving HB and ensuring fire safety requires careful consideration, expertise, and adherence to regulations. Thorough research and seeking professional guidance empower investors to make informed decisions regarding the renovation of HBs that safeguard their cultural significance while minimizing fire risks.

## 6. Conclusion

From an investment perspective, the revitalization of old buildings primarily focused on the potential increase in value. While visible benefits, such as employment opportunities, tax revenue at the project site, and off-site benefits, had been recognized, the invisible values, such as place attachment and cultural identity, also required attention [48–50]. HBs preservation played a crucial role in fostering socioeconomic development, particularly in determining the highest and best use of properties in countries like Egypt [51]. However, if investors did not see a profit, it became increasingly challenging to regenerate old buildings. Despite examining barriers in HB revitalization—like preserving authenticity, meeting modern needs, ensuring fire safety, and maintaining structural integrity—a comprehensive study integrating these challenges was lacking. Existing research often addressed these issues separately, overlooking their interconnected impacts on successful adaptive reuse. Although decision-making in adaptive reuse was explored, balancing stakeholder priorities within a unified framework remained underexplored. This research developed a holistic framework that integrates these challenges, providing practical solutions that consider the interplay of technical and conceptual barriers, guiding practitioners and policymakers toward sustainable and culturally sensitive revitalization.

This research aimed to explore the barriers to the adaptive reuse of heritage buildings for investors and to provide a comprehensive review of the factors influencing adaptive reuse in developing countries. By identifying these factors, this study sought to contribute to the revitalization of HBs, addressing gaps in previous research. Although digital systems for decision-making in investment had become more prevalent, their application to heritage buildings had been limited, often focusing only on intelligent monitoring of preservation status and material conditions. In reality, heritage building investments required consideration of multiple factors, such as the higher renovation costs compared to ordinary buildings. Therefore, understanding these costs in advance was crucial, highlighting the need for more decision-making plrogress. This research aimed to bridge this gap by proposing a more comprehensive AI-driven approach to heritage building revitalization.

This study made several significant contributions to the field of HB revitalization. First, it introduced a correlation analysis based on interviews, demonstrating that vacancy negatively impacted the protection of heritage buildings, whereas rent played a crucial role in safeguarding them. This finding challenged the traditional assumption that vacancy might preserve buildings by limiting wear and tear, instead highlighting the protective value of active tenancy. Second, the research offered a comprehensive review of the factors influencing the adaptive reuse of heritage buildings, particularly in developing countries, and identified key elements that shaped decision-making processes. This review filled a critical gap in existing literature by providing a detailed understanding of the complexities involved in adaptive reuse.

This study enabled the development of a theoretical framework for the integration of collaboration into the HBs revitalization decision-making process including the key factors: (i) investment limitations; (ii) prospect; and (iii) impacts. Through structured interview and field research, the study collected data in the case study area Guangzhou to analyze the attitude of investors for vitalization projects, identify main barriers to revitalize HBs, and see the vacancy as the main damage for the HBs.

**Fig 6:**
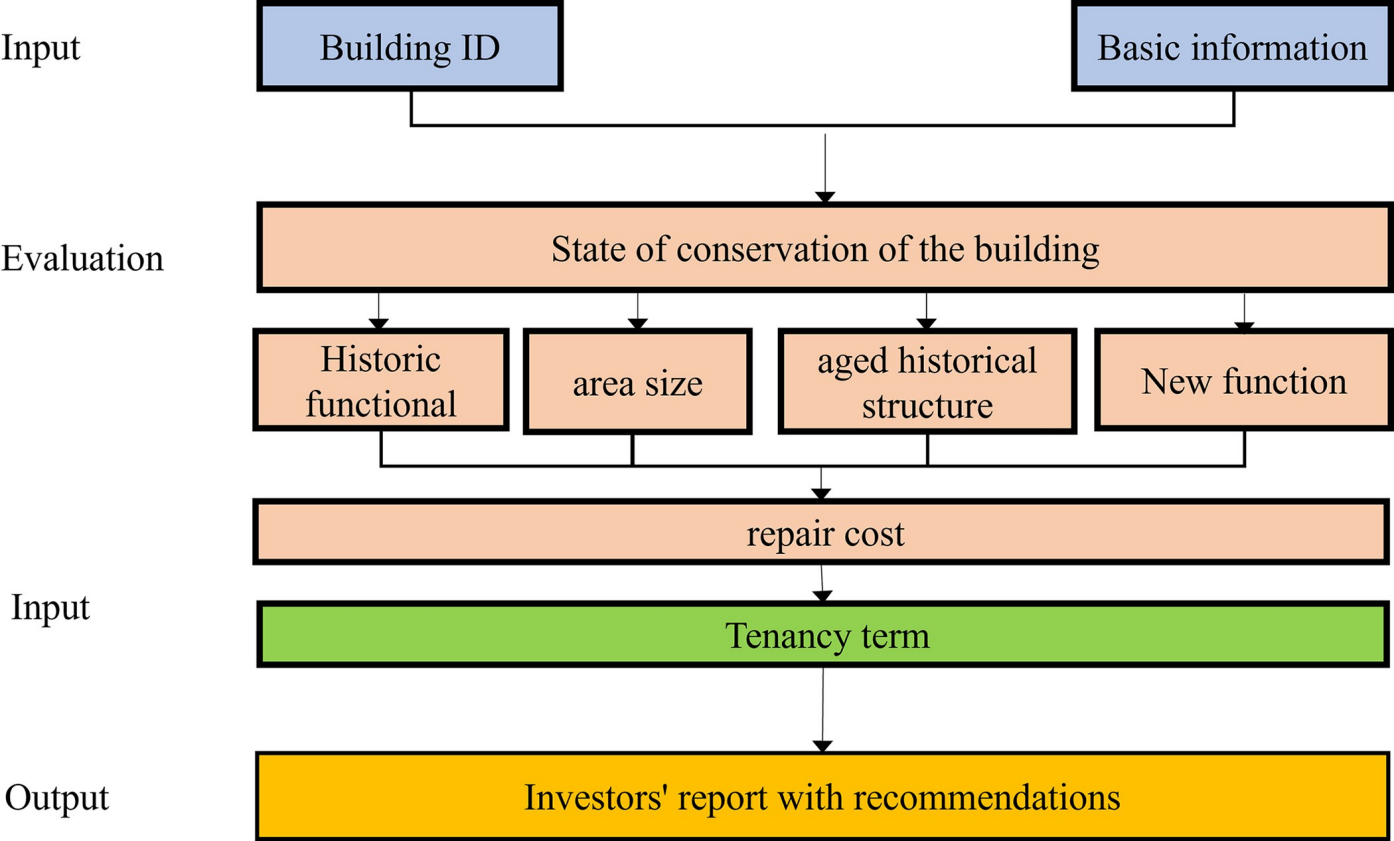
Algorithm framework in revitalizing HBs.

Despite these contributions, the study had limitations that should be acknowledged. The research relied on data collected at a single point in time, which may not have fully captured the dynamic nature of heritage building decline and degradation. While the current research focuses on the key factors affecting the revitalization and adaptive reuse of HBs, there is significant potential for the development of digital decision support systems tailored to heritage building investment. Future work could explore the creation of such systems, which would provide a comprehensive framework for investors to evaluate and compare potential investments in HBs. One possible direction for future research is the development of a system that allows investors to simulate investment scenarios for HBs by assessing a variety of factors, such as functional use (commercial or residential), size, repair costs, and tenancy terms. By employing weighted decision-making models, such systems could offer a more structured and data-driven approach to evaluating the risks and potential returns of heritage building investments. Additionally, integrating a database of HBs, such as the one established for Guangzhou, could enhance decision-making processes by allowing investors to easily access critical data, including building location, age, ownership, and regulatory requirements. This would enable a more efficient assessment of the lease terms, potential functional conversions, and compliance with fire prevention and safety standards. To support better decision-making, normalization techniques could be used to standardize factors such as repair costs and tenancy terms, ensuring comparability across different properties. By automating the generation of transformation proposals, such a system could offer tailored recommendations that align with both investor goals and the preservation needs of the heritage assets. Although AI-based approaches may eventually be integrated, the immediate focus should be on developing practical, digital decision support systems that assist in investment assessments and enhance the adaptive reuse process for HBs. Future studies could refine and test these digital tools in real-world scenarios, contributing to more sustainable and informed decisions in the heritage building sector (See Fig 6).
